# IVUS-Guided Stent Implantation to Improve Outcome: A Promise Waiting to be Fulfilled

**DOI:** 10.2174/157340309788166697

**Published:** 2009-05

**Authors:** Renata Rogacka, Azeem Latib, Antonio Colombo

**Affiliations:** 1 Interventional Cardiology Unit, Desio Hospital, Milan, Italy; 2 Division of Cardiology, Department of Medicine, University of CapeTown, South Africa; 3 Interventional Cardiology Unit, San Raffaele Scientific Institute, Milan, Italy; 4 Interventional Cardiology Unit, EMO-GVM Centro Cuore Columbus, Milan, Italy

**Keywords:** Intravascular ultrasound, drug-eluting stents, stent malapposition, stent underexpansion, percutaneous coronary intervention, restenosis, stent thrombosis.

## Abstract

The use of intravascular ultrasound (IVUS) to improve acute angiographic results was already shown in the prestent era. Various studies demonstrated the efficacy of IVUS in balloon sizing and estimating the extent of positive remodeling. With the introduction of drug-eluting stents (DES) the rate of restenosis has been significantly reduced but a new concern, the risk of stent thrombosis, has emerged. The association of stent underexpansion with stent thrombosis was observed for bare metal stents (BMS) and DES. Until now, the criteria for IVUS optimization used in different studies have relied on distal reference or on mean reference vessel for stent or postdilatation balloon sizing. Furthermore, an important recent innovation not available in previous studies is the use of noncompliant balloons to perform high pressure post-dilatation. Universal and easily applicable IVUS criteria for optimization of stent implantation as well as randomized studies on IVUS-guided DES implantation are necessary to minimize stent malapposition and underexpansion, which in turn can positively influence the rates of stent restenosis and thrombosis.

## INTRODUCTION

The introduction of drug-eluting stents (DES) has reduced the rate of restenosis and the need for repeat revascularization in comparison to bare metal stents (BMS) [1-4]. The promise of stents with minimal or almost no restenosis were eagerly awaited and rapidly adopted by the interventional cardiology community. It was initially believed that the strong antirestenotic effects of the antiproliferative drug coatings would be sufficient to eradicate the problem of in-stent restenosis that had plagued BMS. However, as DES implantation was extended into more complex lesion and patient subsets than previously studied in large randomized trials [1-3, 5], it became clear the optimal DES implantation may be as important if not more with these new devices. The efficacy of DES appears to be related not only to the combination of drug and polymer kinetics but also to how well the device is deployed in the coronary artery. Suboptimal stent implantation (in particular stent underexpansion) has become recognized as an important risk factor not only for DES failure (restenosis) but also for the more serious and rare event of stent thrombosis [6, 7]. 

### Why IVUS-guided stenting?

The use of intravascular ultrasound (IVUS) to improve lesion dilation and the acute angiographic result was already shown in the pre-stent era. Various studies demonstrated the efficacy of IVUS in balloon sizing and estimating the extent of positive remodeling [8]. Small observational studies in the BMS era suggested the benefits of a larger stent cross-sectional area (CSA) and higher postdilation pressures with IVUS guidance, on better stent apposition and restenosis rates [9-12]. Until now, the criteria for IVUS optimization used in different studies have relied on distal vessel reference or on the mean reference vessel size for stent or postdilatation balloon sizing. This reduces the potential to optimally increase the lumen size in long lesions, overlapping stents and in vessels with distal tapering. In addition, these criteria do not take advantage of vessel remodeling which may allow the operator to attain a larger final stent CSA. Also the actual results and differences in final minimal lumen diameter (MLD) achieved with IVUS compared to angiographic guidance has been small in previous studies. Furthermore, an important recent innovation not available in previous studies that may be having a significant contribution on optimal stent implantation is the use of noncompliant balloons to perform high pressure postdilatation. Up to now, there is no randomized study on IVUS-guided DES implantation. Consequently, IVUS guidance has played a limited role in the current clinical practice due a lack of clear demonstrable clinical benefit. IVUS guided stent implantation with modern noncompliant balloons and improved criteria for optimal stent expansion would probably result in an immediate result of better stent expansion and implantation that should translate into a long-term clinical benefit. To test this hypothesis, a new multicenter randomized trial, the Angiographic Versus IVUS Optimization (AVIO), has been designed by our group.

### Previous Trials on IVUS-Guided Stenting

A comparison of the most important studies on IVUS published before the stent era and after the introduction of BMS and DES, may be give a better understanding of the diversity and lack of precision of the IVUS criteria most commonly used for optimal stent implantation. It is interesting to notice a continuous change of IVUS criteria both for balloon sizing and optimal result of percutaneous transluminal coronary angioplasty (PTCA) with or without stent implantation. Honing of the technique and increasing understanding of the information obtainable from ultrasound exploration of coronary arteries were reflected in modifications of IVUS criteria which matured with the experience of the operators. A continuous increase in acute lumen gain has been observed in several randomized and observational studies on coronary stenting. This reflects the learning curve of optimizing stent implantation and expansion which was triggered by the concept “the bigger the better” and the need for additional imaging modalities to avoid stent thrombosis and improve long-term outcomes [13].

Numerous studies have clearly established that a major determinant of the rate of restenosis was the % diameter stenosis (DS) or MLD achieved after intervention, which resulted in the "bigger is better" doctrine [14, 15]. However, attempts to improve the procedural results of PTCA by the use of balloons larger than the angiographic vessel diameter have resulted in unacceptably high rates of major arterial dissection, myocardial infarction, and emergent bypass surgery [16]. The rate of these complications was significantly reduced by the use of IVUS.

In the **Clinical Outcomes with Ultrasound Trial (CLOUT) trial **[8], IVUS was used to guide the selection of balloons traditionally considered oversized on the basis of the degree of plaque burden and vessel expansion in the target lesion and adjacent reference segments. IVUS was performed after balloon angioplasty to establish MLD and minimal vessel diameter (MVD) and calculate the balloon size for an extra dilatation after balloon angioplasty according to the formula: [MLD + MVD] * 0.5 (mm). If the size of the balloon used for the angioplasty was already comparable to the size calculated according to the IVUS-based formula, no further dilatation was necessary. The median increase of the balloon size was 0.5 mm. A significant decrease in the mean angiographic DS was observed after IVUS balloon “oversizing” compared to angiographic evaluation (18±14%* vs. *28±15%), with a corresponding increase in angiographic minimal lumen area (MLA, 1.95±0.49 to 2.21±0.47) and IVUS MLA (3.16±1.04 to 4.52±1.14 mm^2^, p<0.001). The incidence of angiographic dissection was not increased by IVUS-guided balloon upsizing (37%* vs. *40%, p=0.67), emphasizing the role of IVUS guidance in the selection of the "oversized" balloon diameter.

The CLOUT study criteria for balloon sizing were also used in the single-center, prospective randomized **Strategy and Intracoronary Ultrasound-Guided PTCA and Stenting (SIPS) trial **[17]. IVUS measurements were done after PTCA (without stent implantation in 50.3% of the IVUS* vs. *50.5% of the angio-guided group, p=0.96). Even though no significant differences were observed in the primary endpoint (MLD at 6-month angiographic follow-up: 1.71±0.09 for IVUS* vs. *1.56±0.9 mm for angio-guided, p=0.19), the acute postprocedural result (i.e. acute gain) was significantly improved (acute gain in the IVUS 1.85±0.72* vs. *1.67±0.76 mm in the angio-guided group, p = 0.02). This acute improvement in MLD was associated with lower events, and in particular a reduction in clinically driven target lesion revascularization (TLR) at 2 years in the IVUS guided group (21%* vs. *43%, p = 0.02).

In 1995, our group [13] documented by IVUS that majority of stents were inadequately expanded despite an optimal angiographic final result. After IVUS optimization, the intra-stent MLA increased from 6.6 to 8.8 mm^2^. Similarly, other small observational trials of Albiero *et al.* [9] and Blasini *et al.*[10] documented the usefulness of IVUS in assessing appropriate stent expansion.

Optimal stent apposition and expansion had been already pointed out by numerous authors [9, 10, 13] as factors that might improve acute and long-term outcomes after stent implantation, but a clear IVUS definition was lacking. The observational **Multicenter Ultrasound Stenting in Coronaries (MUSIC) trial **[11] was the first study that established IVUS criteria for optimal stent implantation (see Table **1**). IVUS was utilized to assess complete apposition of the stent in all its length against the vessel wall as well as its symmetric expansion. Moreover, numerical criteria, expressed in % of MLA for the reference segment or minimal MLA in mm^2 ^(see Table **1**) were created and subsequently followed in other IVUS-guided stenting studies. In comparison with the BENESTENT I [14] and BENESTENT II [18] studies, the data from the MUSIC trial confirmed that IVUS-guided stent implantation improved immediate angiographic outcomes (post-MLD, 2.90±0.36) which, in turn might explain the favorable clinical and angiographic outcomes at 6 months (the lowest TLR 5.7% and restenosis rate 9.7% with the largest MLD 2.12±0.67). A unique feature of MUSIC was that patients were treated only with single antiplatelet therapy with aspirin (no dicumarols or thienopyridines) after stent implantation, despite which stent thrombosis was documented in only 2 patients (1.3%).

In the **Balloon Equivalent to Stent (BEST) Study **[19], IVUS-guided balloon angioplasty was randomly compared with systematic stenting. A total of 132 patients were randomized to IVUS-guided balloon angioplasty, and 122 were randomized to stenting. In the aggressive PTCA group, crossover to stent was needed in 58 patients (44%) because of dissection (18%) or an unsatisfactory result (26%). The balloon diameter used for aggressive PTCA was determined on the basis of IVUS measurements of mean vessel diameter (determined by external elastic membrane). The balloon inflation followed the compliance curve to obtain the desired diameter. The final MLD was larger in the stent group (2.75±0.49* vs. *2.55±0.49 mm with aggressive PTCA, p=0.013), but patients from the aggressive PTCA group who crossed over to stent had similar immediate results as those in the stent group (2.69±0.49* vs. *2.75±0.49 respectively, p=0.65). At 6-months, the restenosis rate was 16.8±6.7% in the aggressive PTCA group* vs. *18.1±7.0% in the stent group (p=0.70). Also angiographic MLD did not differ significantly between the study groups (1.97±0.72* vs. *2.03±0.72 mm, PTCA and stent respectively, p=0.38). Two other prospective observational studies evaluated an IVUS-guided PTCA-provisional stenting strategy [20, 21]. Abizaid *et al. *[20] enrolled 280 patients, utilizing IVUS for balloon sizing (media-to-media diameter as a criterion) and identifying optimal PTCA results (≥65% of the mean proximal and distal reference lumen areas or ≥6.0 mm^2^ and no dissection). At 1-year follow-up, TLR was necessary in 8% of the PTCA group and 16% in the crossover to stent group. For this reason IVUS-guided aggressive PTCA was proposed as an alternative to routine elective stenting. In the study of Colombo *et al.*[21], 101 consecutive patients with long lesions were treated with IVUS-guided PTCA and spot stenting and confronted with a matched group of patients treated with traditional stenting. Also in this study, the balloon sizing was based on IVUS measurement of media-to-media diameter, while optimal PTCA result was defined by MLD ≥5.5 mm^2^ or ≥50% MVD at the lesion site. Spot stenting was performed only in the segments where the above criteria were not satisfied. The total stent length was significantly lower in the spot stenting group (10.4±13 mm* vs. *32.4±13 mm in the matched group routinely stented, p<0.05) and associated with better long-term outcome (major adverse cardiac events [MACE] 22%* vs. *38%, p<0.05 and angiographic restenosis 19% vs.34%, p<0.05). 

IVUS guidance of stent implantation started being considered an effective aid in adequate stent expansion compared with angiographic guidance alone. This hypothesis was once again confirmed by the IVUS multicenter prospective observational substudy, **Can Routine Ultrasound Influence Stent Expansion (CRUISE) **[12], of the Stent Anti-thrombotic Regimen Study. The most important limitations of the CRUISE trial are that it lacked clear criteria for IVUS optimization and constituted part of a larger study. Nine centers were prospectively assigned to IVUS-guided stent deployment and 7 centers to angiographic guidance alone with documentary (blinded) IVUS at the conclusion of the procedure. The IVUS-guided group had a larger MLD (2.96±0.55* vs. *2.59±0.43, p<0.001) and a larger minimal stent CSA (7.78±1.72* vs. *7.0±62.13 mm2, p<0.001). At 9-months follow-up, TLR occurred significantly less frequently in the IVUS-guided group (8.5%* vs. *15.3%, p<0.05; relative risk reduction of 44%).

De Feyter *et al.* [22] constructed with multivariate logistic regression analysis a reference chart to predict the expected in-stent restenosis (ISR) rate based on operator-dependent IVUS parameters. In-stent MLA (inversely related) and stent length (directly related) were found to be the strongest predictors of ISR at 6 months, confirming once more the “bigger is better” doctrine, which were further confirmed in subsequent randomized trials on IVUS guidance.

The first randomized trial designed to support this hypothesis, **Restenosis after IVUS-guided Stenting (RESIST) study**, was conducted in France by Schiele *et al.* [23]. Even though there was not a significant absolute reduction in the restenosis rate (6.3%; 28.8%* vs. *22.5%, p=0.25), a difference in post-procedural MLD (2.46±0.46 in angiography* vs. *2.57±0.41 in IVUS-guided group, p=0.11) and stent CSA (6.89±2.71 in angio* vs. *7.17±2.48 in IVUS guided group, p=0.35) were observed in this study. Thus a beneficial effect of IVUS guidance could not be ruled out as a result of the small number of the patients included in the study (155) and the lack of statistical power. A significant increase in 6-month stent lumen CSA was observed (4.47±2.59* vs. *5.36±2.81 mm^2^, p=0.03), indicating that IVUS guidance in stent deployment may be beneficial. In the multivariate logistic regression analysis, post-procedural stent CSA was the only predictor (inversely related) of 6-months ISR (odds ratio 0.7, 95% CI 0.47-0.93, p=0.007).

**Optimization with Intracoronary Ultrasound to Reduce Stent Restenosis (OPTICUS) **[24] was a large multicenter study in which 550 patients were randomized to stent implantation with IVUS or angiographic guidance. The MUSIC study criteria were applied for optimal IVUS-guided stent implantation, even though these were achieved only in 56% of patients and the mean stent area in the IVUS group was relatively low (8.1±2.3 mm^2^). Six-months repeat angiography revealed no significant differences between the groups with respect to binary restenosis rates (24.5%* vs. *22.8%, p=0.68) and MLD (1.95±0.72* vs. *1.91±0.68 mm, p=0.52), respectively in the IVUS and angiography-guided groups. At 12 months, neither MACE (relative risk [RR], 1.07; 95% CI 0.75-1.52; p=0.71) nor repeat revascularization (RR 1.04; 95% CI 0.64 to 1.67; p=0.87) were reduced in the IVUS-guided group. The authors concluded that the study does not support routine use of IVUS to guide coronary stenting and the additional procedural costs incurred with IVUS may be saved without exposing patients to excessive risks. However, this conclusion should be interpreted taking into consideration the low percentage of procedures with optimal stent implantation according to established IVUS criteria.

Contrary conclusions, i.e. improved clinical and angiographic outcome in patients treated with IVUS-guided stent implantation, were drawn from the **Thrombocyte Activity Evaluation and Effects of Ultrasound Guidance in Long Intracoronary Stent Placement (TULIP)** study [25]. In this multicenter randomized trial, long lesions (≥20 mm) were analyzed. The IVUS optimization criteria were slightly less aggressive in comparison with the MUSIC trial (See Table **1**) and were reached in 89% of patients. Significant differences were documented between the groups in favor of IVUS-guided stent implantation: greater final MLD (3.01±0.40* vs. *2.80±0.31 mm, p=0.008) and acute gain (2.04±0.62* vs. *1.81±0.45mm, p=0.045), lower binary restenosis rate at 6-months angiographic follow-up (23%* vs. *46%, p=0.0082) and a subsequent decrease in TLR (4%* vs. *14%, p=0.037). Despite MLD at follow-up being significantly larger in the IVUS group (1.82±0.53* vs. *1.51±0.71, p=0.042), there was no difference in late loss (1.20±0.51* vs. *1.33±0.55 in the angiography-guided group, p=NS). 

**The Angiography Versus Intravascular Ultrasound- Directed (AVID) **[26, 27] trial was the largest multicenter randomized study (800 patients) that showed a significant clinical and angiographic benefit of IVUS-guided stenting over angiographic evaluation alone. The first attempt to simplify the IVUS criteria for optimal stent placement was done, in order that these criteria could be applied in everyday interventional practice (see Table **1**). However, according to these criteria, stents were optimally implanted only in 48% of cases. Moreover, only 37% of patients who did not meet IVUS criteria received an additional treatment (e.g. postdilatation for an underexpanded stent). Final stent MLA was 6.90±2.43 mm^2^ in the angiography group and 7.55±2.82 mm^2^ in the IVUS group (p=0.001), results which are comparable with previous reports (see Table **1**). After excluding all the lesions with diameter <2.5 mm, a significant reduction in TLR was observed in the IVUS group at 12 months follow-up (4.3%* vs. *10.1%; p=0.01). Apart from an evident advantage of IVUS guidance over angiography alone in obtaining larger MLD, thus decreasing the need of repeat revascularization, this study raised an important problem of underutilization of IVUS information due to both the lack of experience in evaluating IVUS images by an operator (discrepancy between the measurements done during the procedure and off-line) and a concern about postdilation with larger diameter balloons at higher pressures.

The IVUS studies in the BMS era provided critical insights into the pathophysiology of subacute stent thrombosis and contributed to the modern idea of IVUS optimization of stent implantation. However, despite numerous attempts, no uniform criteria were established for an appropriate IVUS-guided PTCA. Postdilatation was not a standard step after stent implantation and, if performed, the balloon diameter was chosen on the basis of complicated numerical formulas derived from IVUS measurements. In fact, only in a small percentage of the patients treated in the above studies, an adequate postdilatation was performed. Moreover, the IVUS criteria for optimal stent implantation were detailed only in the MUSIC trial (used also in the SIPS and OPTICUS studies). All the other studies utilized less complex criteria, which contributed to simplification of the procedure but resulted in inappropriate stent postdilatation. Moreover, even simplified criteria of IVUS optimization were satisfied only in small percentage of patients, which once again confirms the hypothesis that theoretically acceptable definitions may be inapplicable in everyday practice. 

The question of adequate stent implantation becomes even more important with DES, especially in the era of complex, multivessel and/or left main coronary artery stenting. IVUS should unquestionably help the operator to minimize the risk of stent thrombosis resulting from stent malapposition or underexpansion. To obtain this, simple and easily applicable criteria which do not influence negatively the time of the procedure need to be elaborated. 

### IVUS-Guided Stenting in the DES Era

With the introduction of DES the rate of restenosis has been significantly reduced but a new concern, the risk of stent thrombosis has emerged. Small observational studies demonstrated the association of stent underexpansion with stent thrombosis in BMS [6, 7, 28, 29]. Alfonso *et al.* [30] documented severe stent malapposition in 4 out of 12 patients (33%) presenting with stent thrombosis in BMS. The MUSIC criteria for IVUS optimization were not satisfied in any of them during the index procedure and the stent expansion varied from 41% to 71% (MLA 3.3 to 7.8 mm^2^). 

The past two years have highlighted concerns regarding the safety and efficacy of DES, in particular the increased risk of stent thrombosis and the associated morbidity and mortality [7, 31]. An angioscopic study by Kotani *et al.* [32], demonstrated that more unepithelialized stent struts could be found after DES implantation compared to with BMS at 6 months follow-up. Even though the exact mechanism of thrombus formation in DES is still being investigated, inadequate apposition of the stent within the coronary artery leaving free struts in the lumen may play an important role in thrombogenesis. The definition and pathogenesis of malapposition needs to be clarified for a better understanding of its clinical significance and importance. Incomplete stent apposition (ISA) or stent malapposition is the IVUS finding of lack of contact between stent struts, not overlying a side branch, and the underlying arterial wall [28]. Late ISA can either be due to persistence of acute ISA occurring at the time of stent implantation or late-acquired ISA occurring between stent implantation and follow-up. Traditionally the term “Late ISA” has mainly been used to describe late-acquired ISA. Acute ISA is mostly technique dependant and may be due to suboptimal stent implantation or by severely calcified lesions not allowing for homogeneous stent expansion and resulting in localized stent underexpansion and ISA [6, 33]. In contrast, the pathogenesis of late ISA includes: a) positive arterial remodelling so that the vessel pulls away from the stent; b) dissolution of plaque and thrombus behind the stent, so that a gap forms between the stent and vessel wall; and c) chronic stent recoil [6, 33, 34]. While there does appear to be a link between acute ISA and early (acute and subacute) stent thrombosis, the link between late ISA and late stent thrombosis is not that clear. Late ISA is common, occurring in 10-20% of DES [6, 29, 34], and the majority of late ISA detected on IVUS is not associated with clinical events [28]. As acute malapposition (and underexpansion) are technique-dependant they are correctable if found on IVUS during the procedure. In late-acquired ISA, on the other hand, the post-intervention IVUS demonstrates complete stent apposition and there are no specific factors IVUS that would predict that ISA will develop during the follow-up period. Hong *et al.* [34] observed that ISA occurred in up to 12% of cases of DES implantation, and was more frequent in long stents, chronic total occlusions and stenting during acute myocardial infarction. According to Okabe *et al.* [35], small stent CSA (<4.6mm^2^) and residual disease at stent edges were also predictors of stent thrombosis. 

Waksman *et al.* [36] recently published the data from the largest registry on IVUS-guided PCI with DES. The outcomes in 884 patients (1296 lesions) who underwent IVUS-guided DES implantation to all treated lesions were compared with those in 884 propensity-score matched patients (1312 lesions) who underwent DES implantation with angiographic guidance alone. At 30 days and at 12 months, a higher rate of definite stent thrombosis was seen in the No-IVUS group (0.5* vs. *1.4%; p = 0.046) and (0.7* vs. *2.0%; p = 0.014, respectively). There were no major differences in late stent thrombosis and MACE (14.5* vs. *16.2%; p = 0.33) at 12 month follow-up between the groups. Rates of death and myocardial infarction were similar. A trend was seen in favor of the IVUS group in TLR (5.1* vs. *7.2%; p=0.07). IVUS guidance was an independent predictor of freedom from cumulative stent thrombosis at 12 months (adjusted hazard ratio = 0.5, 95% CI 0.1–0.8; p = 0.02).

Further randomized studies are necessary to establish the exact role of late malapposition in thrombogenesis and IVUS-guided optimal stent expansion in reducing binary restenosis and need of repeat revascularization [28]. 

The introduction of virtual histology intravascular ultrasound (VH-IVUS) may provide new information about the composition of the plaque, correlating it to angiographic and clinical events associated with stent implantation. Kawaguchi *et al.* [37] found VH-IVUS useful in predicting the risk of distal embolization, assessed as ST-segment re-elevation (STR), after stent implantation during primary angioplasty. Necrotic core volume was significantly higher in the STR group (32.9±14.1 mm^3^) than in the non-STR group (20.4±19.1 mm^3^; p<0.05). The similar findings but in a non-acute onset were reported in the paper of Kawamoto *et al.* [38]. The liberation of small embolic components form the necrotic core of the plaque detected and quantified with VH-IVUS, resulted in poorer recovery of coronary flow velocity reserve. However, the clinical utility of VH-IVUS in preventing clinical events still remains to be proven.

## MODERN IVUS CRITERIA

A wide variety of non-uniform IVUS criteria for the optimization of stent implantation has resulted in a large variability in the frequency of achieving various IVUS criteria based on differences in vessel size, plaque burden, and balloon-to-artery ratio, as pointed out by Moussa *et al.* [39]. According to their evaluation of 425 patients, achieving in-stent MLA≥90% of distal reference MLA did not lead to a reduction in restenosis, while reaching an MLA≥9 mm^2^ was associated with a low restenosis rate. This criterion, however, was primarily accomplished in large vessels. Moreover, in-stent MLA≥55% of average reference vessel CSA predicted a higher probability of freedom from restenosis independently from vessel size. The restenosis rate was lower (11%) with MLA≥9mm^2^ and reached 27% with lower MLAs.

Universal and easily applicable IVUS criteria for optimization of stent implantation could resolve, or at least minimize, the problems of stent malapposition and underexpansion, which in turn could positively influence the rates of stent restenosis and thrombosis. Until now, the criteria for IVUS optimization used in different studies have relied on distal reference or on mean reference vessel for stent or postdilatation balloon sizing. This reduces the potential to optimally increase the lumen size particularly in long lesions with overlapping stents and in vessels with distal tapering. Also the actual results and differences in final MLD achieved with IVUS compared to angiographic guidance have been small in previous studies. Furthermore, an important recent innovation not available in previous studies that may be having a significant contribution to optimal stent implantation is the use of noncompliant balloons to perform high pressure postdilatation. Up to now, there is no randomized study on IVUS-guided DES implantation. Consequently, IVUS guidance has played a limited role in the current clinical practice due a lack of clear demonstrable clinical benefit.

Our group is presently supporting the effort to establish modern, universal criteria for IVUS optimization of stent implantation. Our hypothesis is that IVUS guided stent implantation with modern noncompliant balloons and better criteria for optimal stent expansion would result in an immediate result of better stent expansion that will translate into a long-term clinical benefit. The **Angiographic Versus IVUS Optimization (AVIO)** multicenter randomized study, designed by our group, may provide important insights into the importance of IVUS-guided stent implantation in the DES era. The aim of the study is to determine whether IVUS-guided DES implantation in complex lesions is superior to angiographically-guided implantation in improving post-procedural minimum lumen diameter (MLD) as the primary endpoint. The study uses modern criteria for IVUS optimization based on the achievement of a CSA inside the stent corresponding to the area achieved in our preliminary experience, called the achievable optimal result (AOR) in Table **1**. The AOR depends on the diameter of the optimal postdilatation balloon chosen based on the vessel media-media size at different points. These criteria take into account the varying vessel size in overlapping stents and that multiple post-dilations with different size non-compliant balloons may be needed. The flow chart for the AVIO study has been summarized in Fig. (**1**).

We assessed the safety and feasibility of these criteria in a pilot study, PRAVIO: (Preliminary Investigation to the Angiographic Versus IVUS Optimization Trial). For this study, we defined optimization as achieving ≥ 70% of the CSA of the post-dilation balloon. 

The size of this balloon was calculated using the median vessel media-to-media diameters at different sites in the stent segment. The CSA of the stent was measured at the most narrowed zones and stent underexpansion was defined as CSA below 70% of the postdilating balloon that matched the median vessel-vessel diameter at the underexpanded zone. The stent was then postdilated with a non-compliant balloon at any site where the stent CSA was below the 70% criteria, and if needed several different sized balloons were used in long-overlapping stents where the vessel diameter varied in size. Using these criteria, we were able to achieve a minimal stent CSA ≥ 70% of the postdilation balloon in 78% of lesions that underwent IVUS optimization. We then compared the final MLD in 93 IVUS optimized lesions with a group of angiographically-guided lesions matched according to diabetes, vessel type, reference vessel diameter (RVD), MLD and lesion length. Baseline RVD, MLD and lesion lengths were not statistically different between the two matched groups. However, final MLD was significantly larger in the IVUS compared to the angiographic guided group (3.09 ± 0.50 v 2.67 ± 0.54; p < 0.0001). From a practical point of view, we noted that despite a similar baseline angiographic RVD, the use of IVUS gave the operator the confidence to select a larger postdilating balloon size (3.26 ± 0.50* vs. *2.98 ± 0.42 mm; p<0.001) and safely inflate the balloon to higher pressures (24.4 ± 4.6* vs. *16.1 ± 5.0 atmospheres; p<0.001), Fig. (**2**). 

Interestingly, from this pilot study we found that using the “>70% CSA of postdilating balloon” criteria was an underestimation of the actual result that we were achieving and thus redefined our AOR. Thus for the randomized AVIO trial, the criteria that will be utilized (Table **2**), uses the AOR we found in this pilot study.

## CONCLUSIONS

The search for clear and uniform criteria for IVUS optimization is still ongoing, especially in the DES era. Even more importantly, whether IVUS-guided DES implantation will have benefits on clinical outcomes remains unproven. The growing need to lower the risk of DES thrombosis and restenosis coupled with the treatment of more complex lesions may give IVUS-guided stenting another opportunity to prove itself. The results and clinical applicability of the AVIO trial to routine clinical practice may shed some light on whether the promise of IVUS-guided stent implantation has a role in the DES era.

## Figures and Tables

**Fig. (1):**
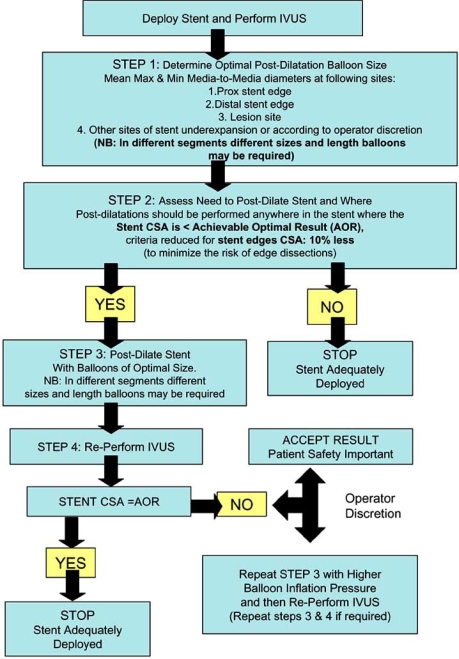
AVIO study flow chart.

**Fig. (2):**
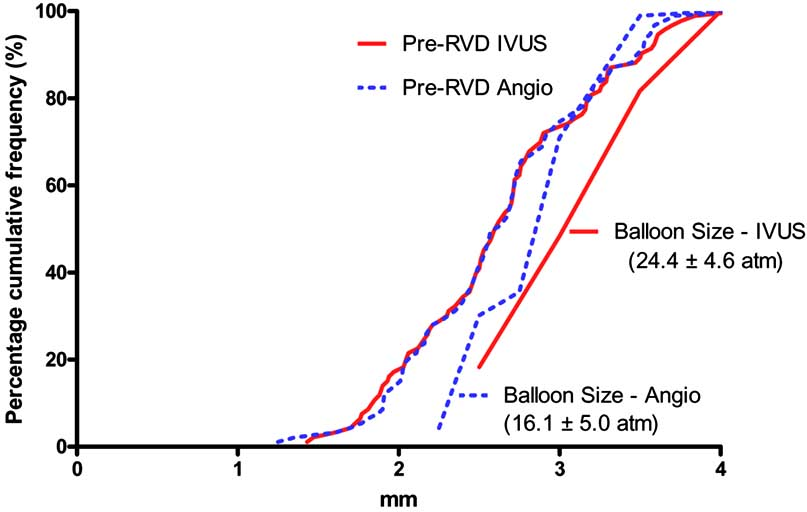
Cumulative frequency curves for baseline RVD and final ballon size in matched lesions optimized with IVUS versus angiography.

**Table 1:** Comparison of Studies with IVUS Optimization of Percutaneous Transluminal Coronary Angioplasty (PTCA) with or without Stent Implantation

	Balloon Sizing (BS)	IVUS Criteria	Results		Study Design
			Angiographic	IVUS	
**CLOUT [8] 1997**	(MLD+MVD)*0.5 in the proximal and distal segment	If PTCA balloon size was already equal to BS obtained according to the formula, no further dilatation performed (27% lesions)	**MLA ** 2.21±0.47 **Acute gain **0.20	**MLA ** 4.52±1.14, p<0.001 **Acute gain ** 0.26, p<0.001	155 pts; observational study; only postprocedural endpoints
**MUSIC [11] 1998**	IVUS used for evaluation of appropriate stent apposition.	Complete apposition against the vessel wall of the entire stent. MLA≥90% of the average reference lumen area or ≥100% of lumen area of the reference segment with the lowest lumen area. MLA >9.0 mm2. MLA ≥80% of the average reference lumen area or ≥90% of lumen area of the reference segment with the lowest lumen area. Symmetric stent expansion.	**MLD** 2.90±0.36; **Acute gain ** 1.79±0.39	**MLD ** 3.10±0.40	161 pts; observational
			7 mo TLR: 5.7%		
**SIPS [17] 2000**	(MLD+MVD)*0.5 in the prox e distal segment		**MLD ** 2.38±0.67; **Acute gain** 1.67±0.76	**MLD ** 2.49±0.66, p=0.12; **Acute gain ** 1.85±0.72; p=0.02	269 pts, randomized prospective; 6 mo angiographic and 2 years clinical FU
			2 years clinical FU: clinically driven **TLR **		
			43%	21%, p=0.02	
**CRUISE [12] 2000**	No detailed IVUS criteria	Post procedure IVUS analysis in the core lab	**MLD** 2.59±0.43 **MLA** 7.06±2.13	**MLD**, 2.96±0.55, p<0.001 **MLA ** 7.78±1.72	525 pts, multicenter prospective observational IVUS substudy
			9-month FU		
			**TLR ** 15.3%	**TLR ** 8.5%, p< 0.05	
**BEST [19] 2003**	Balloon diameter closest to the vessel diameter (EEM mean diameter)	IVUS criteria for crossover to stent: >30% stenosis or MLA< 6 mm^2^	IVUS-guided PTCA	Stent	254 pts, multicenter, randomized
			**MLD** 2.55±0.49 **MLA**6.60±2.05	**MLD ** 2.75±0.49, p=0.013 **MLA ** 7.28±2.22, p=0.02	
			6-mo FU: no significant angiographic and MACE differences		
**RESIST [23] 1998 **		Stent CSA>80% of the mean proximal and distal reference vessel CSA *Only 77% of pts with adequate angiographic result satisfied IVUS criteria *	**MLD ** 2.46±0.46 **Acute gain ** 1.45±0.53 **MLA ** 7.16±2.48	**MLD ** 2.57±0.41 **Acute gain ** 1.62±0.43, p=0.04 **MLA ** 7.95±2.21, p=0.04	155 pts; multicenter, randomized, single-blinded
			6-months FU		
			**Restenosis **28.8% **MLA **4.47±2.59	**Restenosis **22.5%, p=0.25 **MLA ** 5.36±2.81, p=0.03	
**OPTICUS [24] 2001**		MUSIC criteria for optimal stent implantation *achieved in only 56% of patients *	**MLD**2.91±0.41 **Acute gain ** 1.91±0.66	**MLD** 3.02±0.49; p=0.01 **Acute gain**2.07±0.50; p<0.0001 **MLA **8.10±2.30	550 pts; multicenter, randomized
			6-months FU		
			**Binary restenosis ** 22.8% **Late loss** 1.00±0.58	**Binary restenosis ** 24.5%, p=0.68 **Late loss**1.07±0.62; p=0.20	
**TULIP [25] 2002**		complete stent apposition; MLD≥80% of the mean of prox and distal reference diameters; MLA≥ distal reference lumen area. Criteria accomplished in 89% of patients	**MLD** 2.80±0.31 **Acute gain** 1.81±0.45	**MLD ** 3.01±0.40, p=0.008 **Acute gain ** 2.04±0.62, p=0.045 **MLA ** 6.00±3.30	150 pts, multicenter, randomized
			6-months FU		
			**Binary restenosis ** 46% **Late loss**1.33±0.55 ** TLR ** 10%	**Binary restenosis ** 23%, p=0.008 **Late loss**1.20±0.51, p=NS **TLR ** 3%, p=0.037	
**AVID [26, 27] 2000**		MLA ≥90% of distal minimal vessel lumen CSA; Stent fully apposed; Dissections covered by stent Criteria accomplished only in 48% patients	**MLD** 2.89±0.51 **MLA** 6.90±2.43	**MLD ** 2.95±0.49, p=0.15 **MLA ** 7.55±2.82, p=0.001	800 pts, multicenter randomized
			12 months FU		
			**TLR**10.1%	**TLR**4.3%, p=0.01	
**AVIO **	median vessel media-to-media diameters at different sites in the stent segment	AOR (see Table **2**)	ongoing multicenter randomized trial		

IVUS- intravascular ultrasound; MLD- minimal lumen diameter (mm), MLA- in stent minimal lumen area (mm^2^); TLR- target lesion revascularization; FU- follow-up; EEM-external elastic membrane; AOR- achievable optimal result.

**Table 2:** AVIO Study Criteria for Optimal DES Implantation Based on - Achievable Optimal Result (AOR) is the Target Minimum Stent Cross-Sectional Area (CSA) According to the Non-Compliant Balloon Chosen for Postdilatation. The Diameter of the balloon is chosen on the basis of the average Media-to-Media Diameters of the Vessel at Different Points of the Stented Area (Proximal, Mid Lesion, Distal and any other Points of Interest such as Point of Maximum Underexpansion)

Balloon Size (mm)	Achievable Optimal Result (mm^2^)
2.25	3.5
2.5	4
3	6
3.5	8
4	10
4.5	12
